# Combined Therapy with Intravenous Immunoglobulins, Letermovir and (Val-)Ganciclovir in Complicated Courses of CMV-Infection in Transplant Recipients

**DOI:** 10.3390/microorganisms9081666

**Published:** 2021-08-04

**Authors:** Veronica Di Cristanziano, Patrick Affeldt, Moritz Trappe, Maike Wirtz, Eva Heger, Elena Knops, Rolf Kaiser, Dirk Stippel, Roman-Ulrich Müller, Udo Holtick, Christoph Scheid, Martin Kann, Christine E. Kurschat, Franziska Grundmann

**Affiliations:** 1 Institute of Virology, Faculty of Medicine, University Hospital Cologne, University of Cologne, Fürst-Pückler Straße 56, 50935 Cologne, Germany; veronica.di-cristanziano@uk-koeln.de (V.D.C.); trappem@smail.uni-koeln.de (M.T.); maike.wirtz@uk-koeln.de (M.W.); eva.heger@uk-koeln.de (E.H.); elena.knops@uk-koeln.de (E.K.); rolf.kaiser@uk-koeln.de (R.K.); 2 Department II of Internal Medicine and Center for Molecular Medicine Cologne, Faculty of Medicine, University Hospital Cologne, University of Cologne, Kerpener Straße 62, 50937 Cologne, Germany; patrick.affeldt@uk-koeln.de (P.A.); roman-ulrich.mueller@uk-koeln.de (R.-U.M.); martin.kann@uk-koeln.de (M.K.); franziska.grundmann@uk-koeln.de (F.G.); 3 Department of General, Visceral, Cancer and Transplant Surgery, Faculty of Medicine, University Hospital Cologne, University of Cologne, Kerpener Straße 62, 50937 Cologne, Germany; dirk.stippel@uk-koeln.de; 4 CECAD, Faculty of Medicine, University Hospital Cologne, University of Cologne, Joseph-Stelzmann Straße 26, 50931 Cologne, Germany; 5 Department I of Internal Medicine, Faculty of Medicine, University Hospital Cologne, University of Cologne, Kerpener Straße 62, 50937 Cologne, Germany; udo.holtick@uk-koeln.de (U.H.); christoph.scheid@uk-koeln.de (C.S.)

**Keywords:** CMV, ELISPOT, drug resistance, kidney, bone marrow, transplantation, immunosuppression, opportunistic infection, combination, cellular response

## Abstract

The treatment options for cytomegalovirus (CMV) infections in immunosuppressed patients are limited, mainly consisting of (val-)ganciclovir (VGC/GCV) as the first-line treatment. We report on three transplant recipients, one stem cell transplant (allo-HSCT) patient and two kidney transplant (KTx) recipients, with prolonged CMV viremia treated with a combined therapy based on letermovir (LMV), CMV-specific intravenous immunoglobulins (IVIg), and VGC/GCV, which led to the sustained control of CMV viremia in all patients.

## 1. Introduction

CMV infection represents one of the most important opportunistic viral infections in allo-HSCT and KTx recipients [1,2]. The current first-line prevention and preemptive therapy are based on (val-)ganciclovir (VGC/GCV) [3]. Severe side effects often lead to dose reduction, causing subtherapeutic levels, prolonged therapy duration, and increased risk for the development of drug resistance [2,4]. Additionally, CMV-specific intravenous immunoglobulins (CMV IVIg) are approved as prophylactic therapy in Europe and can be considered as prophylactic treatment option after allo-HSCT and KTx [5].

Since 2018, letermovir (LMV) has also been approved as a prophylactic treatment following allo-HSCT in CMV-seropositive patients [6]. In KTx, the prophylactic use of LMV demonstrated a similar level of effectiveness, but had less severe side effects than the standard-of-care therapy with VGC [7]. Nonetheless, the therapeutic use of LMV remains off-label in allo-HSCT and KTx recipients [8].

Monitoring the cellular CD4+ and CD8+ T-cell response is an upcoming issue after HSCT and KTx [9]. In patients after KTx, low CMV-specific T-cell reactivity was associated with a risk of CMV reactivation at the end of CMV prophylaxis [10]. In addition, low T-cell response against CMV in solid organ transplantation (SOT) and in HSCT was associated with complicated CMV treatment courses, triggering CMV resistance [11,12].

In this case series, we report our single-center experience of a therapeutic combination of LMV, VGC/GCV, and CMV IVIg for complicated courses of CMV infection after allo-HSCT and KT. We also present the diagnostic screening by ELISPOT (enzyme-linked immunospot) using the T-SPOT.CMV assay (Oxford Immunotec, Milton, Oxford, UK) to assess specific cellular immunity to CMV.

The retrospective data collection was approved by the institutional review board (21-1171) and all patients gave their written informed consent.

## 2. Materials and Methods

CMV DNA was detected in whole blood using a GeneProof CMV PCR Kit (Medac GmbH, Wedel, Germany). The PCR for CMV DNA in the blood was carried once per time point and patient together with a positive and negative control to verify the result.

Anti-viral drug resistance testing against VGC/GCV, FOS, CDV, and LMV was performed by amplification and sequencing of UL-97, UL-54, and UL-56. Interpretation was based on the bioinformatic tool MRA (“mutation resistance analyzer”) developed by the Institute of Virology of the University Hospital of Ulm, Germany (https://www.informatik.uni-ulm.de/ni/mitarbeiter/HKestler/mra/app/index.php?plugin=contact, accessed on 29 July 2021).

IFN-γ-producing T-cells (CD4+ and CD8+) reactive against CMV IE-1 and pp65 antigens were measured by the T-SPOT.CMV (IFN-γ release assay, Oxford Immunotec, Milton, Oxford, UK), according to the manufacturer’s instructions.

## 3. Clinical Cases

**Figure 1:**
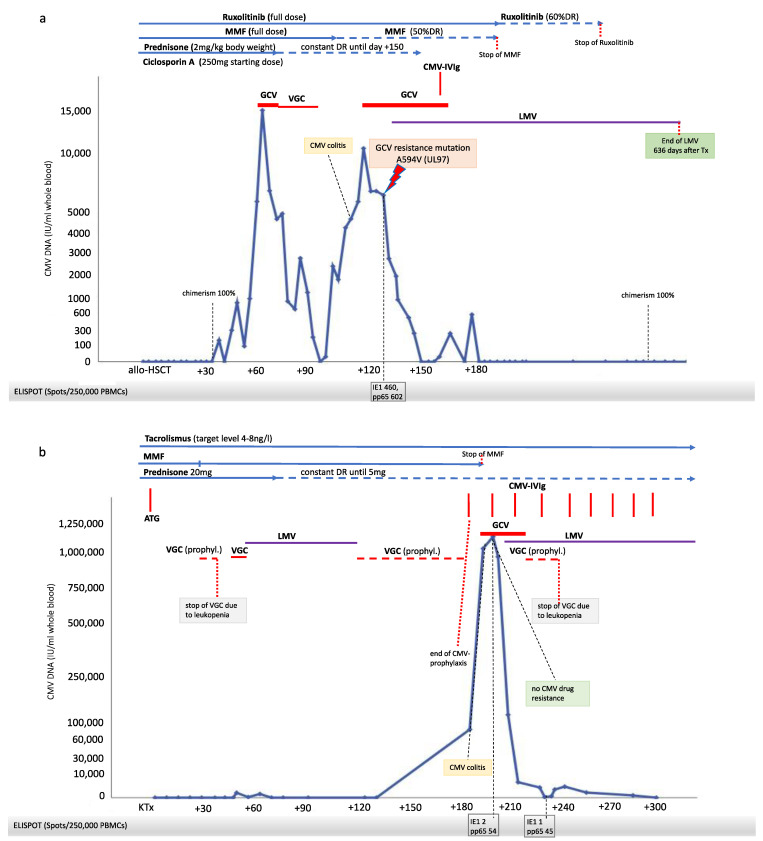

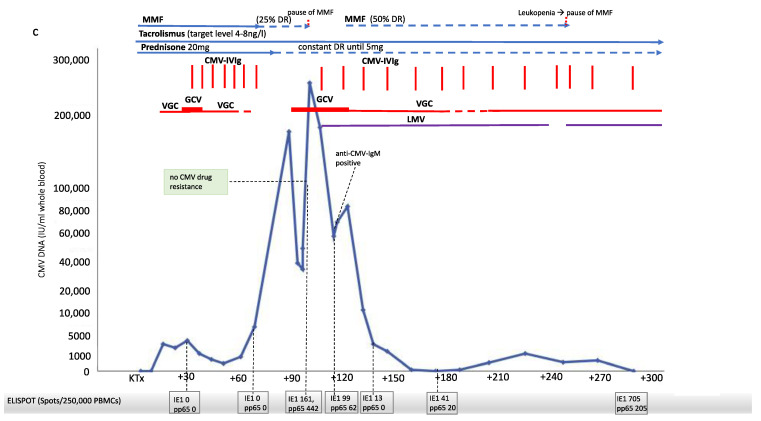
Post-transplantation dynamics of CMV viremia, immunosuppressive therapy, cellular immunity against CMV (T-SPOT.CMV Elispot assay by Oxford Immunotec), antiviral strategies and clinical course in three patients after transplantation. *X*-axis: days after transplantation. (**a**) Case 1. Allogeneic–hematopoietic stem cell transplantation (allo-HSCT) recipient (HLA 10/10 MUD; CMV serostatus D+/R+; ABO compatible). (**b**) Case 2. Kidney transplantation (KTx) from deceased donor (HLA MM A-1, B-2, DR-2; CMV serostatus D+/R−; AB0 compatible). (**c**) Case 3. Kidney transplantation from living donor (HLA MM A-0, B-1, DR-1; CMV serostatus D−/R−; ABO compatible). Abbreviations: ATG, Anti-Thymocyte Globulin; DR, dose reduction; GCV, ganciclovir; CMV-IVIg, CMV-intravenous immunoglobulin; IE1, immediate-early 1; LMV, letermovir; MMF, mycophenolate mofetil; pp65, phosphoprotein 65; VGC, valganciclovir CMV-DNA was detected from whole blood using GeneProof CMV PCR Kit (Medac GmbH, Wedel, Germany). § Anti-viral drug resistance testing against VGC/GCV, FOS, CDV, and LMV was performed by amplification and sequencing of UL-97, UL-54, and UL-56. Interpretation was based on the bioinformatic tool MRA (“mutation resistance analyzer”) developed by the Institute of Virology of the University Hospital of Ulm, Germany (https://www.informatik.uni-ulm.de/ni/mitarbeiter/HKestler/mra/app/index.php?plugin=contact, accessed on 29 July 2021). IFN-γ producing T-cells (CD4+ and CD8+) reactive against CMV IE-1 and pp65 antigens were measured by the T-SPOT.CMV (Oxford Immunotec, Milton, UK), according to the manufacturer’s instructions.

Case 1: A 57-year-old CMV-positive male patient who had been diagnosed with acute myeloid leukemia (AML) underwent allo-HSCT from a matched unrelated CMV-positive donor 4 months after diagnosis. At the time of allo-HSCT, he was in cytomorphological complete remission after 2 cycles of 7 + 3 (cytarabine and daunorubicin) induction and one consolidation cycle with high-dose cytarabine. Under 7 + 3 therapy, the patient suffered from drug-induced toxic acute kidney injury. On day +41 after transplantation, CMV DNA tested positive, and the patient received intravenous GCV, which was replaced by oral VGC after the viral load started decreasing. After the termination of therapy, CMV-associated colitis was diagnosed on day +113 by biopsy and presented clinically as diarrhea. Due to the severity of his CMV end-organ disease, immunosuppression was reduced and the patient was treated again with intravenous GCV; however, an increasing level of CMV viremia was observed. A mutation of UL-97, confirming resistance to VGC/GCV, was detected, but the analysis of the T-cell immune response to CMV by ELISPOT assay showed adequate reactivity. As foscarnet and cidofovir come with an unfavorable safety profile in a patient with acute kidney injury, a combination therapy of intravenous IVIg and oral LMV (480 mg/d) was added to VGC/GCV therapy (which was maintained to prevent LMV resistance) and stable CMV clearance was achieved. LMV was discontinued 636 days after transplantation (Figure 1a).

Case 2: A 48-year-old CMV-IgG- and IgM-negative female received a KTx from a deceased CMV-IgG-positive donor. Shortly after transplantation, the patient was treated with anti-thymocyte globulin and immunoadsorption due to the acute rejection. As the patient had shown progressive leukopenia after KTx even before rejection therapy, VGC therapy was discontinued and CMV PCR was monitored weekly. In the further course, leukopenia normalized and prophylactic therapy with VGC was re-initiated but had to be paused after 3 days because of recurrent leucopenia. At week 6 post-KTx, blood screening revealed a positive CMV PCR. The recurrence of leukopenia after initiation of therapeutic VGC therapy led to the decision of changing therapy to LMV (480 mg once daily) and to pause VGC therapy. Stable CMV clearance was gained after 42 days of therapy (two negative PCR tests within two weeks). Therapy was switched back to prophylactic VGC (450 mg/d, adapted to eGFR). One hundred and ninety-one days post-KTx, PCR testing for CMV DNA revealed 77,000 copies, and the patient presented with diarrhea as a clinical symptom of CMV-associated colitis. Due to severe leukopenia, VGC therapy was discontinued, and CMV IVIg therapy was instituted (100 IE/kg body weight); however, the viral load increased to >1,000,000 copies in a few days. Consequently, the patient was hospitalized, immunosuppression with mycophenolate mofetil (MMF) was stopped, and additional treatment with intravenous GCV initiated. Because of the life-threatening clinical situation, rising CMV levels and weak CMV T-cell reactivity by ELISPOT, LMV was introduced again as an add-on therapy. The resistance analysis of CMV against VGC/GCV was negative. A triple combination therapy led to a rapid decrease in CMV viremia. GCV was replaced by oral VGC, but VGC treatment again resulted in leukopenia. Under current medication, consisting of LMV (480 mg) and CMV-IVIg, sustained clearance of CMV viremia was achieved. LMV therapy is continued to date (Figure 1b).

Case 3: A 61-year-old CMV IgG- and IgM-negative female received an AB0-compatible kidney transplant from a living donor in which pre-transplantation CMV IgG and IgM were also negative. Eighteen days post-KTx, the recipient developed a primary CMV infection. At this time point, the CMV status in the donor was assessed again, revealing positive CMV IgG and negative CMV IgM. These data indicated that the donor experienced a primary CMV infection in the interval between pre-KTX screening and transplantation. With positive CMV DNA detection 17 days after transplantation, oral VGC therapy was initiated. During VGC therapy, a rising CMV viral load was observed, and therapy was changed to intravenous GCV therapy. At this time, ELISPOT showed a weak T-cell reactivity to CMV, and CMV IgM was measured negative even though the patient developed CMV IgG. At this time, additional therapy with CMV IVIg was initiated. The combined therapy led to reduction in CMV load, VGC was reintroduced, and the patient was discharged with VGC/CMV IVIg therapy. Seven days later, laboratory testing revealed rising CMV copies. CMV ELISPOT showed no reactivity to CMV; therefore, MMF was reduced by 25%. Within one week, CMV copies increased to 170,000 IU/mL and the patient presented with fever, diarrhea and deterioration of general condition. The patient was hospitalized to resume GCV treatment and to continue CMV IVIg therapy. MMF was paused, and, as GCV resistance was suspected, LMV was added to the therapeutic regimen, leading to a rapid decrease in CMV copies. Resistance testing remained negative, but ELISPOT still showed a weak T-cell reactivity to CMV. Repeated testing of T-cell reactivity to CMV indicated an improvement of cellular immunity over time. After 30 days, GCV was replaced by VGC, and MMF was restarted in reduced dose (50%). After 56 days of triple therapy (VGC, LMV + CMV IVIg), CMV replication could be controlled and, finally, CMV viremia was cleared. LMV therapy is continued to date (Figure 1c).

## 4. Discussion

CMV infections are a major complication after allo-HSCT and KTx, adversely affecting patient outcomes. The treatment of CMV still poses a challenge to treating physicians due to the side effects of available drugs and the occurrence of CMV drug resistance [1,2].

In kidney transplant recipients with an intermediate-to-high risk of contracting CMV infections, VGC has been established as a prophylactic and preemptive treatment and is still the first line-therapy for CMV infections after allo-HSCT and KTx [3,5].

The dose reduction in immunosuppressive treatment should be evaluated in patients suffering from CMV infection to strengthen cellular immunity, but this will come at the risk of graft rejection [5]. Following the reduction in MMF therapy, only a weak CD4+ and CD8+ T-cell response to CMV was detected in both KTx recipients, both suffering from primary CMV infection following transplantation. A weak cellular immunity against CMV has been associated with primary failure of VGC therapy, prolonged therapy duration, and higher CMV relapse rates [11,12]. In both KTx patients, no CMV antiviral drug resistance was detected, whereas early relapse of CMV infection in the allo-HSCT recipient was likely associated with a resistance mutation against VGC/GCV detected in the viral phosphotransferase gene UL97.

There are limited data classifying CMV T-cell reactivity in immunosuppressed patients. In the literature, >20–40 spots/250,000 lymphocytes (against IE1 and pp65) in patients after KTx were associated with a lower risk for CMV infection [10,13]. Spots <20/250,000 may, therefore, be classified as weak cellular immunity. Assuming that ELISPOT is suitable as an indicator for quantifying the cellular immune response to CMV, fluctuations in the number of spots can be interpreted as a result of improvement of functionality in the cellular immune system. As shown in case 3, after pausing MMF therapy due to severe leukopenia, ELISPOT showed T-cell reactivity in this patient for the first time. After reintroducing MMF therapy, T-cell reactivity was weaker again, and recurrence of CMV viremia occurred despite ongoing anti-CMV triple therapy.

In recent years, the use of LMV has been established as prophylactic treatment option after allo-HSCT in patients with high risk for CMV infection [6]. The potential use of LMV in the therapeutic management of CMV infections is being increasingly discussed, and the first therapeutic experiences have been reported [14]. Nevertheless, LMV therapy remains off-label in the therapeutic setting of allo-HSCT patients, and in general in patients after KTx. Its use in clinical management of post-transplant CMV infections can be restricted to CMV recurrence and development of drug resistance [5,15]. The impairment of the immune system due to immunosuppression related to transplantation procedures may compromise the patient’s capacity to control CMV replication, especially when the recipient becomes infected by CMV in the allograft. Increasing amounts of data demonstrated that the assessment of CMV cellular immune response represents a useful predictive parameter to more effectively stratify the risk of CMV infection or reactivation in transplant recipients [10,16].

After the introduction of HIV therapy, similar problems occurred and finally led to the use of current antiretroviral therapy protocols (ART), employing a combination of drugs against different viral targets [17]. Using this idea of multi-drug antiviral therapy to establish a reliable virus clearance, we decided to start a combination of VGC or GCV/LMV/CMV IVIg therapy in our patients. In combination therapy, CMV replication was controlled in all patients. These data underline the beneficial effects that multi-target antiviral therapy have in patients with persistent CMV infection after transplantation, limiting the occurrence of severe side effects.

In a previous case, we reported that LMV monotherapy in immunocompromised patients can cause the rapid development of resistance [15]. In that case, the LMV resistance variant occurred in the wild-type background, despite the previous replication of a GCV-resistant viral strain, and the GCV resistance was no longer detectable. Based on these previous observations, the combined use of LMV and GCV, despite the previously observed GCV resistance in the allo-HSCT recipient, was intended to keep genetic pressure on the less replication-competent GCV-resistant viral strain.

Our case series describes the potential benefit of using of a multi-target anti-CMV therapy in case of persistent CMV viremia associated with poor antiviral cellular immunity to CMV.

## 5. Conclusions

In patients with evidence of drug resistance against first-line anti-viral treatment and/or low CMV CD4+ and CD8+ reactivity by ELISPOT, a combination of VGC/GCV + LMV + CMV IVIg therapy should be considered. The optimal time of therapy initiation and optimal CD4+ and CD8+ reactivity for the successful return to prophylactic VGC treatment remain unclear and further prospective studies are needed to evaluate these aspects.

## Data Availability

Data supporting reported results is available upon request.
